# Sicherheit und Monitoring der patientenkontrollierten intravenösen Analgesie: Anwendungspraxis in deutschen Krankenhäusern

**DOI:** 10.1007/s00101-020-00907-2

**Published:** 2020-12-29

**Authors:** M. I. Emons, M. Maring, U. M. Stamer, E. Pogatzki-Zahn, F. Petzke, J. Erlenwein

**Affiliations:** 1 Klinik für Anästhesiologie, Universitätsmedizin Göttingen, Robert Koch Str. 40, 37075 Göttingen, Deutschland; 2 Klinik für Anästhesiologie und Schmerztherapie, Inselspital, Universität Bern und Department of BioMedical Research, Universität Bern, Bern, Schweiz; 3 Klinik für Anästhesiologie, operative Intensivmedizin und Schmerztherapie, Universitätsklinikum Münster, Münster, Deutschland; 4 Arbeitskreis Akutschmerz, Deutsche Schmerzgesellschaft e. V., Berlin, Deutschland; 5 Wissenschaftlicher Arbeitskreis Schmerzmedizin, Deutsche Gesellschaft für Anästhesiologie und Intensivmedizin e. V., Nürnberg, Deutschland

**Keywords:** Akutschmerzdienst, Opioide, Atemdepression, Risikomanagement, Medikamentenverwechslungen, Acute pain service, Opioids, Respiratory depression, Risk management, Medication errors

## Abstract

**Hintergrund:**

Die patientenkontrollierte intravenöse Analgesie („patient controlled intravenous analgesia“, PCIA) ist als Verfahren in der Akutschmerztherapie etabliert. Ziel dieser Untersuchung war es, Anwendungspraxis, Überwachung, unerwünschte Vorkommnisse und Komplikationen unter einer PCIA-Therapie an deutschen Krankenhäusern zu erfassen.

**Methoden:**

Alle 995 bei der Deutschen Gesellschaft für Anästhesie und Intensivmedizin e. V. registrierten Chefärzte wurden zur Teilnahme an der elektronischen Umfrage eingeladen.

**Ergenisse:**

Aus 244 Kliniken wurden Antworten zurückgesandt. In 193 (79 %) dieser Kliniken kam die PCIA zum Einsatz. Alle folgenden Angaben beziehen sich auf diese Krankenhäuser. Das am häufigsten genutzte Opioid war Piritramid. Bei Patienten mit PCIA setzten 94 % der Kliniken zusätzlich Nichtopioidanalgetika ein, 38 % retardierte orale Opioide sowie 4 % parenterale Opioide. Bei Anwendung der PCIA auf Normalstation lag lediglich in 31 % der Kliniken ein standardisiertes Überwachungskonzept vor, das über die Routineversorgung der Stationen hinausging. Insgesamt 82 % der befragten Kliniken berichteten von unerwünschten Vorkommnissen im Zusammenhang mit der PCIA. In 39 % der Kliniken war in den vorangegangenen 6 Monaten mindestens eine potenziell vital bedrohliche Komplikation aufgetreten, insgesamt wurden 335 Einzelfälle berichtet (bei ca. 50.000 durchgeführten PCIAs). Kliniken, die über Komplikationen berichteten, hatten einen höheren Überwachungsstandard als Kliniken, die über keine Komplikationen berichteten.

**Schlussfolgerungen:**

Die PCIA ist ein verbreitetes, aber durchaus mit Risiken verbundenes Analgesieverfahren. Anwendungs- und Überwachungspraxis sind heterogen. Konsentierte, aktuelle Empfehlungen hinsichtlich Behandlungs- und Überwachungsstandards sowie der systematischen Erfassung von Komplikationen bei Anwendung der PCIA stehen aus.

## Abstract (en)

**Background:**

Patient-controlled intravenous analgesia (PCIA) is a well-established technique in acute pain management and available in most German hospitals. Despite its widespread use, information on current clinical practice is limited. This investigation evaluated clinical practice and monitoring as well as PCIA-associated adverse events and critical incidents in German hospitals.

**Methods:**

An invitation to participate in this online-survey was sent to 995 heads of anesthesiology departments belonging to the “German Society of Anaesthesiology and Intensive Care Medicine”.

**Results:**

Of the departments receiving the link, 244 took part (response rate 25%). PCIA was used in 193 of these hospitals (79%). All the following statements relate to the hospitals in which PCIA was used. Piritramide was the most frequently used opioid. In parallel with PCIA, additional nonopioid analgesics were used in 94% of the hospitals, and in 38%, additional slow-release oral opioids were used. Parenteral opioids were administered by the ward staff in 4% of the hospitals. In 75% of hospitals, there were standardized indications for PCIA therapy, with almost two thirds of respondents stating that PCIA was the technique of second choice if regional procedures were contraindicated or failed. In all, 76% of the hospitals had an acute pain service. Twenty-four percent of the hospitals regularly used PCIA in non-surgical patients. In pediatric patients, PCIA was used in 62 hospitals (32%). Only 31% of the hospitals reported the use of standardized protocols for the specific monitoring of patients’ vital signs on general wards, exceeding general care. Of the department, 158 (82%) reported adverse events in connection with the use of PCIA within the six-month period preceding the survey (most frequently due to patients’ noncompliance [52%], dislocated intravenous lines [41%], communication errors [16%], administration of additional analgesics [16%] and/ or sedatives [14%], problems with the pump [16%], programming errors [9%], incorrect opioid concentration in the reservoir [8%], non-observance of contraindications [7%], incorrect dosing [6%] and self-dosing by the patient [4%] or by third parties [3%], filling the reservoir with thewrong medication [2%]; and other problems [5%]). Only 35 of the hospitals (18%) reported no problems associated with PCIA therapy. Seventy-five of the 193 respondents (39%) stated that at least one critical incident had occurred in the context of the use of PCIA. This resulted in a total of 335 cases out of an estimate of 50.000 patients treated with PCIA. The respondents classified these as follows: I) 273 incidents requiring a prolonged stay in the recovery room, but without further complications, II) 58 requiring transfer to the intensive care unit, but without further complications, III) three resulting in permanent harm to the patient and IV) one resulting in the death of the patient. A comparison of the monitoring standards for PCIA showed that critical incidents were reported less frequently in hospitals with less intensive monitoring, and more frequently in hospitals with higher monitoring standards.

**Conclusion:**

PCIA is a frequently used analgesic technique in German hospitals. There were many differences in how PCIA therapy was applied and monitored on general wards. Adverse events occurred to a significant extent, with a considerable part of them, which might be preventable. Critical incidents were perceived more often when standards for monitoring on general wards were higher. Consented current recommendations regarding treatment and monitoring standards as well as the systematic recording of complications when using PCIA are pending.

## Hintergrund

Seit der Entwicklung und Einführung der patientenkontrollierten intravenösen Analgesie („patient controlled intravenous analgesia“, PCIA) in die klinische Praxis in den 1970er-Jahren [14], hat sich die PCIA als Verfahren in der Akutschmerztherapie etabliert [19]. In den meisten deutschen Krankenhäusern ist dieses Analgesieverfahren verfügbar [9]. Das Prinzip beruht darauf, dass sich ein Patient entsprechend voreingestellter Parameter selber i.v.-Boli eines Opioids verabreichen kann [20]. Einprogrammierte Sperrzeiten und Maximaldosen für bestimmte Zeitintervalle sollen zur Sicherheit dieser Applikationstechnik beitragen. Weiterhin wird argumentiert, dass bei zu hoher kumulierter Opioiddosis eine einsetzende Sedierung des Patienten zu einer Abnahme weiterer Bolusapplikationen führt, damit also das Risiko für eine schwere Atemdepression reduziert werden kann.

Die häufigsten unerwünschten Ereignisse der PCIA sind opioidtypische Nebenwirkungen wie Übelkeit und Erbrechen, seltener Pruritus und Harnverhalt [8]. Als potenziell vital bedrohliche Komplikationen können dosisabhängig zentralnervöse Nebenwirkungen wie Verwirrtheitszustände, eine zunehmende Sedierung bis hin zu einer Atemdepression auftreten. Einzelfallberichte und Analysen von Zwischenfällen lassen darauf schließen, dass insbesondere eine Komedikation mit sedierenden oder atemdepressiven Substanzen, Fehlprogrammierungen der PCIA-Pumpe und eine zusätzliche kontinuierliche i.v.-Opioid-Applikation („Basalrate“) zu Opioidüberdosierungen mit Zwischenfällen und Tod des Patienten führen können [3, 19, 21, 33].

Alle Verfahren der Akutschmerztherapie, seien es Techniken der Regionalanalgesie oder eine systemische Analgesie, tragen gewisse Risiken in sich [5, 26]. Trotz tragischer Einzelfälle sowie durch von Laien angestoßene Kampagnen für ein intensives Monitoring der PCIA in Nordamerika besteht Unklarheit, wie die PCIA im klinischen Alltag zur Gewährleistung der bestmöglichen Patientensicherheit eingesetzt und überwacht werden soll [1, 3, 23, 25, 26]. Trotz der weiten Verbreitung des Verfahrens der PCIA fehlen Daten zur Anwendungspraxis in der klinischen Routine.

Fragestellung: Ziel dieser Untersuchung war es, die Anwendungspraxis und das Monitoring der PCIA-Therapie an deutschen Krankenhäusern zu erfassen. Außerdem wurden das Auftreten unerwünschter Vorkommnisse und potenziell vital bedrohlicher Komplikationen erfragt, die in der klinischen Routineanwendung beobachtet worden waren.

## Methode

Die Datenerfassung dieser Befragung erfolgte anhand eines elektronischen Fragebogens mit dem Befragungstool Survey Monkey (SurveyMonkey Inc, Palo Alto, Kalifornien, USA, 2015). Bei der DGAI registrierte Chefärzte (Mitgliederverzeichnis 15.06.2015) erhielten am 23.06.2015 per Mail eine Einladung zur Teilnahme sowie den Link zur Befragung. Am 06.07.2015 wurde eine Erinnerungs-Mail versandt. Der Link konnte jeweils nur einmal pro Adressat bearbeitet werden und wurde bei Datenextraktion am 31.07.2015 geschlossen. Zur Verbesserung des Rücklaufs wurde unter den teilnehmenden Abteilungen ein Preis verlost (2 Teilnahmeplätze zum „Akutschmerzkurs“, Deutsche Schmerzgesellschaft e. V. Berlin).

## Fragebogen

Der Fragebogen wurde in einem mehrstufigen Verfahren entworfen: Zunächst erfolgte ein Brainstorming zu Themen und Aspekten unter den Autoren. Die Angaben wurden thematisch sortiert und zusammengefasst. Daraus wurde ein Fragebogen entworfen, der in einem Probelauf mit 10 weiteren Kollegen auf Inhalt und Verständlichkeit geprüft und anschließend überarbeitet wurde. Der endgültige Fragebogen umfasste:

- **allgemeine Angaben zum Krankenhaus**: Versorgungsstufe, Bettenzahl, Trägerschaft, Vorhandensein und Charakteristik eines Akutschmerzdienstes (ASD),
- **Anwendungspraxis der PCIA**: jeweils getrennt erfasst für Erwachsene und Kinder (Alter <15 Jahre): Anwendungshäufigkeit innerhalb der letzten 6 Monate, eingesetzte Opioide, Pumpenprogrammierung, Medikamentenkombinationen, Einsatzgebiete, Indikationen und Kontraindikationen, Verantwortlichkeit.
- **Monitoring: **Vorhandensein von Überwachungsstandards und entsprechende Abfrage von Überwachungsmodi (kontinuierliche bzw. intermittierende Pulsoxymetrie, Sedierungsgrad anhand eines Scores, Atemfrequenz, Atemmuster),
- **Patientensicherheit:**
  1. allgemeine Probleme in der Anwendungspraxis, bezogen auf den Zeitraum der vorangegangenen 6 Monate (abgefragte Problematiken: Kommunikationsprobleme, Manipulation der Pumpeneinstellung durch den Patienten, dislozierter venöser Zugang, technische Probleme der PCIA-Pumpe, Überdosierung durch Betätigung des Anforderungsbuttons durch Dritte, Missbrauch durch Diebstahl/Entnahme von Opioiden durch Dritte, Nichtbeachten von Kontraindikationen, falsches Medikament, inkorrekte Medikamentendosis oder Programmierungsfehler der Pumpe, Patientenverwechselung, parallele Gabe anderer zentral wirksamer Medikamente [z. B. Sedativa], zusätzliche Gabe anderer nichterwünschter/-indizierter Analgetika, mangelnde Compliance oder Überforderung der Patienten mit der PCIA, Sonstiges),
  2. aufgetretene unerwünschte Vorkommnisse und potenziell vital bedrohliche Komplikationen, bezogen auf den Zeitraum der vorangegangenen 6 Monate, Ursachen, Häufigkeit und Schweregrad (Grad I: „folgenloser Aufenthalt im Aufwachraum/oder besondere Nachbeobachtung auf Allgemeinstation“; Grad II: „folgenlose Verlegung auf Intensiv- oder Wachstation“; Grad III: „bleibender Schaden des Patienten“; Grad IV: „Tod des Patienten“).

## Statistik

Die Darstellung der Ergebnisse erfolgte primär deskriptiv mittels SPSS Statistics (Version 24 IBM, Armonk, USA). Prozentangaben wurden gerundet. Zusammenhänge zwischen Überwachungsmodi und berichteten vitalen Komplikationen wurden mittels χ2-Test nach Pearson dargestellt (Signifikanzniveau *p* ≤ 0,05). Eine Adjustierung des Signifikanzniveaus bei Mehrfachvergleichen erfolgte aufgrund des primär deskriptiven Charakters dieser Untersuchung nicht.

## Ergebnisse

### Rücklauf

Von den 995 Adressaten, die einen Link für die Onlinebefragung erhielten, füllten 244 Kolleginnen und Kollegen den Fragebogen aus (Rücklauf 25 %). Die folgenden Ergebnisse beziehen sich auf die 79 % (*n* = 193) der Antwortenden, in deren Kliniken die PCIA als Verfahren genutzt wurde. 9 % (*n* = 22) der Antwortenden gaben an, keine PCIA zu nutzen, in weiteren 12 % (*n* = 29) wurden hierzu keine Angaben gemacht und diese Krankenhäuser somit von der Analyse ausgeschlossen. Die Angaben werden, wenn nicht anders benannt, für erwachsene Patienten dargestellt. Die Ergebnisse für die Nutzung des Verfahrens bei Kindern und Jugendlichen sind in einem separaten Abschnitt dargestellt.

Die meisten Antworten kamen aus Krankenhäusern der Regel- und Schwerpunktversorgung, im Vergleich zur Verteilung der Trägerschaft in Deutschland überdurchschnittlich häufig aus Kliniken freigemeinnütziger Träger (Tab. 1, zum Vergleich: Grunddaten für Krankenhäuser in Deutschland: private Trägerschaft 37 %, freigemeinnützige Trägerschaft: 34 %, öffentlicher Trägerschaft 29 % [6]). Von diesen Kliniken verfügten 76 % (*n* = 147) über einen Akutschmerzdienst; kein ASD war in 24 % (*n* = 46) der Kliniken etabliert.

**Table 1:** 

*Versorgungsstufe*	*% (n)*
Grundversorgung	14 (28)
Regel‑/Schwerpunktversorgung	51 (99)
Maximalversorgung	23 (44)
Fach- und Belegklinik	11 (21)
Sonstiges	1 (1)
*Trägerschaft*	*% (n)*
Öffentlich	38 (74)
Freigemeinnützig	40 (78)
Privat	22 (41)
*Bettenzahl*	*% (n)*
≤199	26 (50)
200–399	33 (63)
400–699	26 (51)
700–999	5 (9)
≥1000	10 (20)

## Anwendungshäufigkeit

Nach Angaben der Befragten wurde innerhalb des Bezugszeitraums von 6 Monaten in deren Kliniken insgesamt bei ca. 50.000 Patienten eine PCIA-Therapie durchgeführt. 56 Kliniken gaben mit insgesamt 13.202 behandelten Patienten exakte Zahlen an (Mittelwert 236 Patienten/Krankenhaus, durchschnittlich 9 Patienten/Woche). Die anderen Kliniken schätzten ihre Patientenzahl mit PCIA-Therapie zusammen auf insgesamt 39.402 Fälle (Mittelwert 279 Patienten/Krankenhaus, durchschnittlich 11 Patienten/Woche).

## Indikationen und Kontraindikationen

In 75 % der Krankenhäuser bestanden festgelegte Indikationen für eine PCIA-Therapie: Knapp zwei Drittel gaben an, eine PCIA zu nutzen, wenn ein regionales Analgesieverfahren (RA) kontraindiziert war, die Anlage eines RA misslang oder mit der RA keine suffiziente Analgesie erzielt werden konnte. In 17 % der Kliniken war die PCIA für bestimmte Eingriffe (z. B. Endoprothetik) als analgetisches Standardverfahren festgelegt. Definierte Kontraindikationen für den Einsatz einer PCIA wurden für 60 % der Krankenhäuser angegeben (Angaben beziehen sich auf die Krankenhäuser mit definierten Kontraindikationen, Mehrfachantworten möglich): Schlafapnoesyndrom (38 %), Zustand nach (23 %) oder aktueller Drogenabusus (32 %), COPD (6 %), Adipositas per magna (6 %) sowie Incompliance (3 %), Demenz bzw. kognitive Einschränkungen (4 %). Die Indikationsstellung zur PCIA erfolgte am häufigsten in den meisten Krankenhäusern durch den Anästhesisten im Aufwachraum (72 %), durch den betreuenden Anästhesisten im OP (67 %), durch den Anästhesisten bei der Prämedikation (49 %) oder durch den ASD (48 %), seltener durch den Stationsarzt (15 %) oder den Operateur (12 %; Mehrfachantworten möglich).

## Medikamente und Pumpeneinstellungen

Am häufigsten wurde zur PCIA-Therapie das Opioid Piritramid eingesetzt, gefolgt von Oxycodon und Hydromorphon (Tab. 2). In 88 % der Krankenhäuser war jeweils nur ein einzelner Wirkstoff als Standardopioid vorgesehen, in 12 % wurden regelmäßig unterschiedliche Opioide für die PCIA genutzt. Piritramid war mit Abstand das Opioid der ersten Wahl (81 % der Kliniken). Deutlich seltener wurden Oxycodon (14 %), Morphin (2 %), Hydromorphon (2 %) oder Fentanyl (1 %) als Opioid der ersten Wahl eingesetzt. Eine Kombination mehrerer Medikamente im PCIA-Reservoir erfolgte in 15 % der Krankenhäuser: Antiemetika wurden in 10 %, Metamizol in 7 % und Ketamin/Ketanest in 1 % mit einem Opioid als Pumpenfüllung kombiniert (sonstige Kombinationen 3 %, Mehrfachantworten möglich; keine Medikamentenkombinationen im Reservoir 85 %). In fast allen Krankenhäusern erhielten die Patienten zusätzlich zur PCIA eine Basisanalgesie mit Nichtopioidanalgetika. In 38 % der Kliniken wurden in der Routineversorgung parallel retardierte orale Opioide zusätzlich zur PCIA gegeben (Tab. 2). In wenigen Kliniken wurden zusätzlich zur PCIA regelhaft parenterale Opioide durch die Stationsmitarbeiter appliziert.

**Table 2:** 

*Verwendete Opioide*	*% (n)*^*a*^
Piritramid	88 (169)
Oxycodon	18 (34)
Morphin	19 (37)
Hydromorphon	11 (22)
Fentanyl	2 (3)
Sufentanil	4 (8)
Buprenorphin	1 (2)
Remifentanil	2 (4)
Tramadol	1 (1)
*Zusätzliche Analgesie*	*% (n)*^*a*^
Nichtopioidanalgetika	94 (182)
Orale retardierte Opioide	38 (73)
Zusätzliche parenterale Opioide	4 (7)
Keine anderen Analgetika	2 (4)

^a^Mehrfachantwort möglich

Von den Antwortenden gaben 64 % für ihr Krankenhaus an, dass eine Standarddosis als Bolus für alle Erwachsenen verwendet wird; 29 % programmierten den Bolus jeweils individuell nach klinischer Einschätzung des Anästhesisten bzw. 5 % nach Körpergewicht (Sonstiges: 2 %). In 71 % der Kliniken wurde grundsätzlich auf eine Basalrate verzichtet, in 26 % eine Basalrate ausschließlich in Überwachungsbereichen angewandt. Hingegen wurde eine Basalrate auf allen Stationen, inklusive der Normalstationen, in 9 % der Kliniken genutzt.

## Verantwortlichkeit und Überwachung

Von den Kliniken, in denen eine PCIA angewandt wurde, verfügten 147 (76 %, *n* = 10 fehlende Angabe) über einen ASD (Charakteristika: Tab. 3). Bei Anwendung der PCIA auf Normalstation erfolgte in 34 % der Krankenhäuser neben der routinemäßigen Erfassung allgemeiner Vitalparameter im Rahmen der normalen Stationsabläufe keine zusätzliche Erfassung von Vitalparametern bei PCIA-Therapie. In 35 % der Kliniken erfolgte „bei Bedarf“ eine zusätzliche Überwachung, und in 31 % der Kliniken wurden auf Normalstation die Vitalparameter der Patienten mit PCIA innerhalb der ersten 24 h nach einem festen Zeitschema zusätzlich überwacht. Allgemeine Vitalparameter, v. a. Blutdruck und Herzfrequenz wurden in der Regel von den Pflegenden der Stationen erfasst, deutlich seltener durch den ASD oder, falls kein ASD vorhanden war, durch den Anästhesisten bzw. die Anästhesiepflege. Zusätzlich erfasste Vitalparameter waren die Atemfrequenz und das Atemmuster, die Sauerstoffsättigung durch intermittierende oder kontinuierliche Pulsoxymetrie sowie der Sedierungsgrad anhand eines Scores (Tab. 4). Nebenwirkungen unter einer PCIA-Therapie wurden in 63 % der Kliniken standardisiert erfasst. Es zeigte sich, dass in den Kliniken, in denen bei PCIA-Patienten ein erweitertes Monitoring etabliert war, eine kontinuierliche Pulsoxymetrie in 50 % der Kliniken eingesetzt wurde, jedoch nur in 22 % der Kliniken mit einer stationsüblichen Standardüberwachung (χ^2^ = 12,223, *p* < 0,001). Weiterhin wurde in diesen Kliniken häufiger die Sauerstoffsättigung mittels intermittierender Pulsoxymetrie gemessen (73 % vs. 42 %, χ^2^ = 15,031, *p* < 0,001) sowie die Atemfrequenz (89 % vs. 66 %, χ^2^ = 13,871, *p* < 0,001), das Atemmuster (83 % vs. 53 %, χ^2^ = 18,328, *p* < 0,001), die Herzfrequenz (100 % vs. 90 %, χ^2^ = 8,558, *p* = 0,003), der Blutdruck (100 % vs. 95 %, χ^2^ = 5,848, *p* = 0,016) und der Sedierungsgrad anhand eines Scores (90 % vs. 70 %, χ^2^ = 11,933, *p* = 0,001) erfasst. Keine Unterschiede zeigten sich bei der Erfassung der Nebenwirkungen, der Patientenzufriedenheit, der Urinausscheidung, der Schmerzintensität, der technischen Kontrolle der PCIA sowie der Dokumentation angeforderter und erhaltener Boli.

**Table 3:** 

Ärztliche Besetzung des Akutschmerzdienstes^*^ Kliniken mit ASD	*% (n)*
Ausschließliche Zuständigkeit für Akutschmerztherapie	18 (27)
Schmerzdienst neben anderen Tätigkeiten	78 (114)
Keine ärztliche Besetzung des Schmerzdienstes	4 (6)
*Pflegerische Besetzung des Akutschmerzdienstes*	*% (n)*
Ausschließliche Zuständigkeit für Akutschmerztherapie	39 (57)
Schmerzdienst neben anderen Tätigkeiten	44 (64)
Keine pflegerische Besetzung des Schmerzdienstes	16 (23)
Sonstiges	2 (3)
*Ärztliche Zuständigkeit außerhalb der Regelarbeitszeit*	*% (n)*
Zusätzlicher ärztlicher (Ruf‑)Dienst, der ausschließlich oder überwiegend für den Akutschmerzdienst zuständig ist	8 (11)
Die regulären ärztlichen Bereitschaftsdienste, die den Akutschmerzdienst mit abdecken	83 (122)
Kein Akutschmerzdienst außerhalb der Regelarbeitszeit verfügbar	3 (5)
Außerhalb der Regelarbeitszeiten kein ärztlicher, jedoch pflegerischer Akutschmerzdienst verfügbar	4 (6)
Nicht festgelegt	1 (1)
Sonstiges	1 (2)
*Zuständigkeit eines Oberarztes für die Organisation und Ausbildung im Akutschmerzdienst*	*% (n)*
Ja, ein fester	59 (86)
Ja, mehrere	20 (30)
Nein	21 (31)
*Einbindung des Akutschmerzdienstes in den Rotationsplan zur Weiterbildung der Assistenten (fest für mind. einen Monat)*	*% (n)*
Ja, nur für Assistenten der Anästhesiologie	37 (55)
Ja, für Assistenten der Anästhesie und der operativen Fachrichtungen	2 (3)
Nein	59 (86)
Sonstiges	2 (3)

**Table 4:** 

	Standardisiert erfasst % (*n*)	Erhoben durch % (*n*)^a^					
		Stationspflege	ASD	Anästhesist^b^	Anästhesiepflege^b^	Stationsarzt	Operateur
Intermittierende Pulsoxymetrie	35 (68)	74 (52)	35 (24)	19 (13)	6 (4)	–	–
Kontinuierliche Pulsoxymetrie	13 (25)	85 (22)	23 (6)	15 (4)	8 (2)	–	–
Atemfrequenz	64 (123)	81 (100)	46 (56)	15 (19)	6 (7)	2 (2)	1 (1)
Atemmuster	46 (88)	63 (55)	60 (52)	18 (16)	7 (6)	2 (2)	2 (2)
Schmerzintensität	86 (166)	88 (146)	69 (115)	19 (32)	7 (12)	11 (19)	4 (6)
Sedierungsgrad (anhand eines Scores)	60 (113)	60 (53)	83 (74)	20 (18)	9 (8)	2 (2)	1 (1)

^a^Mehrfachantworten möglich

^b^Nur, wenn kein ASD vorhanden

## PCIA-Therapie bei Kindern

Eine PCIA wurde in 32 % (*n* = 62) der Kliniken bei Kindern und Jugendlichen unter 15 Jahren angewandt. Auch in dieser Patientengruppe wurde am häufigsten Piritramid eingesetzt (95 %). 10 % der Krankenhäuser nutzten zudem regelmäßig ein weiteres Opioid zur PCIA (Oxycodon, Hydromorphon, Morphin). In 6 Krankenhäusern wurde für Kinder bzw. Jugendliche regelhaft eine Basalrate genutzt (immer: *n* = 1, nur in Ausnahmefällen: *n* = 5). Die Überwachung der Kinder mit einer PCIA war heterogen. In 46 % der Krankenhäuser wurden Kinder mit einer PCIA standardmäßig nach einem festen Schema überwacht, während in 35 % zusätzliche Vitalparameter nur nach Bedarf erfasst wurden. Keine Überwachung über die Routineerfassung der Station hinaus wurde von 19 % der Antwortenden angegeben.

## Andere Einsatzbereiche

Der regelmäßige Einsatz der PCIA in nichtoperativen Abteilungen wurde für 24 % der Krankenhäuser angegeben, am häufigsten bei Patienten der inneren Medizin (18 %) und der Palliativmedizin (14 %), vereinzelt auch in der Strahlentherapie (3 %), der Geriatrie (2 %), der Neurologie und bei nichtoperativen Patienten der Pädiatrie (2 %, Mehrfachantworten möglich; andere: 5 %). Durchschnittlich wurden in diesen Krankenhäusern wöchentlich 1–2 konservativ behandelte Patienten mit einer PCIA versorgt.

## Patientensicherheit

Von den befragten Kliniken berichteten 82 % (*n* = 158) für den 6‑monatigen Bezugszeitraum über unerwünschte Vorkommnisse im Zusammenhang mit der Nutzung einer PCIA (Tab. 5). Häufig waren diese patientenbezogen, gefolgt von technischen bzw. verfahrensbedingten Problemen. Die behandlerbezogenen Probleme wurden insgesamt etwas seltener genannt, hatten aber potenziell eine hohe Relevanz für das Outcome.

**Table 5:** 

	Gesamt % (*n*)	Kliniken ohne ASD % (*n*)	Kliniken mit ASD % (*n*)
*Patientenbezogene Probleme*			
Überforderung des Patienten	52 (100)	50 (23)	52 (77)
Eigenständige Dosierung durch den Patienten	4 (7)	0	5 (7)
*Technische/verfahrensbedingte Probleme*			
Dislozierte venöse Katheter	41 (79)	37 (17)	42 (62)
Probleme mit Maschinen‑/Pumpensystemen	16 (31)	9 (4)	18 (27)
Manipulation der Dosierung durch Dritte	3 (6)	4 (2)	3 (4)
*Behandlerbezogene Probleme*			
Kommunikationsfehler	16 (31)	15 (7)	16 (24)
Gabe zusätzlicher Analgetika	16 (30)	11 (5)	17 (25)
Zusätzliche Gabe von Sedativa	14 (26)	9 (4)	15 (22)
Programmierungsfehler	9 (17)	4 (2)	10 (15)
Falsche Opioidkonzentration im Reservoir	8 (15)	7 (3)	8 (12)
Nichtbeachten von Kontraindikationen	7 (14)	9 (4)	7 (10)
Fehldosierungen durch die Behandler	6 (12)	0	8 (12)
Füllung des Reservoirs mit dem falschen Medikament	2 (3)	2 (1)	2 (3)
*Keine Probleme unter PCIA-Therapie*	18 (35)	17 (8)	18 (27)

Zur orientierenden Einschätzung der Patientensicherheit unter einer PCIA-Therapie wurde nach potenziell vital bedrohlichen Komplikationen sowie deren Schweregrad gefragt. Von 39 % der Kliniken (*n* = 75) wurde angegeben, dass innerhalb des Bezugszeitraums mindestens eine potenziell vital bedrohliche Komplikation aufgetreten sei, insgesamt 335 berichtete Einzelfälle. Diese wurden von den Befragten folgendermaßen eingestuft: 273 als Komplikation I. Grades („folgenloser Aufenthalt im Aufwachraum/oder besondere Nachbeobachtung auf Allgemeinstation“), 58 als Komplikationen II. Grades („folgenlose Verlegung auf Intensivstation“), 3 als Komplikationen III. Grades („bleibender Schaden des Patienten“) und ein Fall einer Komplikation IV. Grades („Tod des Patienten“). Für die 4 Fälle mit Grad III und IV wurden keine konkreten Angaben zur Art des Ereignisses gemacht.

**Figure 1:**
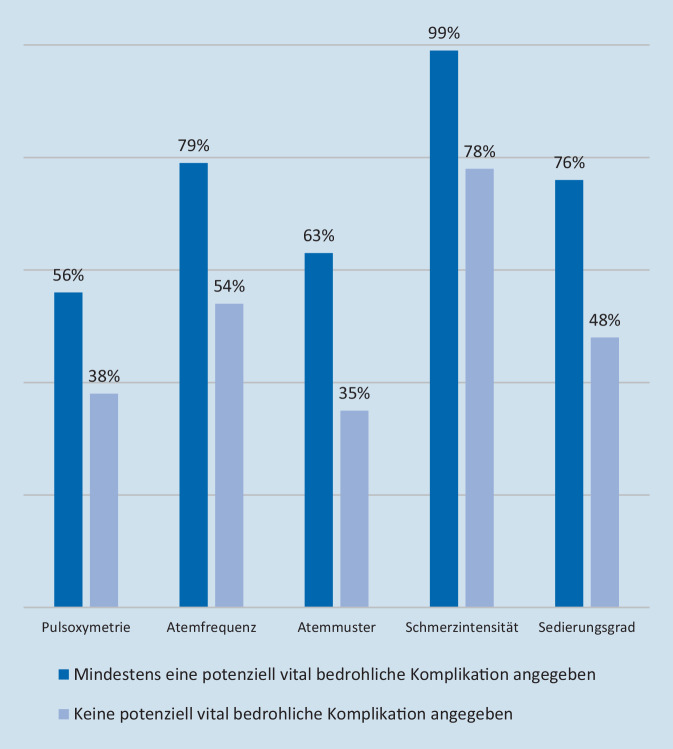

Bei der Gegenüberstellung der Überwachungsmodi der Kliniken zeigte sich, dass in Kliniken, die mindestens eine potenziell vital bedrohliche Komplikation berichteten, eine intensivere Überwachung von Vitalparametern durchgeführt wurde als in Kliniken, aus denen keine Komplikationen berichtet wurden (Abb. 1). War ein ASD vorhanden, wurde von 41 % der Kliniken über potenziell bedrohliche Komplikationen berichtet vs. 31 % in Kliniken ohne ASD (χ^2^ = 0,993, *p* = 0,319). Weiterhin zeigte sich, dass 49 % der Kliniken, in denen regelmäßig retardierte Opioide zusätzlich zur PCIA eingesetzt wurden, über stattgehabte vital bedrohliche Komplikationen berichteten, vs. 33 % in Krankenhäusern, in denen keine retardierten Opioide zusätzlich verabreicht wurden (χ^2^0 = 5,402, *p* = 0,02). Jedoch verfügten diese Kliniken auch signifikant häufiger über einen Akutschmerzdienst (χ^2^ = 8,561, *p* = 0,003), und die PCIA-Therapie wurde häufiger mittels kontinuierlicher Pulsoxymetrie überwacht (χ^2^ = 5,725, *p* = 0,017).

## Diskussion

Die Umfrage zur PCIA diente den Arbeitskreisen als Diskussionsgrundlage zur aktuellen Anwendungspraxis und Überwachungsstandards der PCIA in Deutschland. Trotz mehrerer Reviews zum Thema PCIA fehlen bislang konkrete Informationen zu diesem Thema [2, 24]. Im Anbetracht dieser Wissenslücke und der Diskussion um die Sicherheit der PCIA im Kontext fehlender Überwachungsstandards sind die Ergebnisse aus Sicht der Autoren für die klinische Praxis von großer Bedeutung: Zum einen zeigt diese Umfrage, dass die PCIA ein weit verbreitetes Analgesieverfahren ist und in Krankenhäusern aller Versorgungsstufen zum Einsatz kommt. Andererseits zeigt sich jedoch auch, dass die Praxis der klinischen Anwendung und Überwachung heterogen ist. Dieses mag Folge fehlender, aktueller deutscher Empfehlungen zu Überwachungsstandards sein. Die meisten Kliniken berichteten über unerwünschte Vorkommnisse im Zusammenhang mit der PCIA, die für die Patientensicherheit bedeutend sein können. Nur etwa ein Fünftel der Kliniken, die die PCIA nutzen, gaben an, dass keine solcher Vorkommnisse aufgetreten seien. Zudem berichtete ein relevanter Anteil, dass in den Monaten vor der Befragung potenziell vital bedrohliche Komplikationen im Kontext der PCIA-Therapie aufgetreten seien. Dabei zeigte sich ein Zusammenhang zwischen berichteten Komplikationen und der Überwachungspraxis. Bei intensiverem Monitoring von Vitalparametern wurde häufiger über Komplikationen berichtet, während in Kliniken, die keine unerwünschten Ereignisse berichteten, die PCIA-Therapie weniger intensiv überwacht wurde.

## Repräsentativität und Limitation

Die Ergebnisse konnten mit einer Erfassung von ca. 15 % der deutschen Krankenhäuser einen wichtigen Einblick in die Anwendungspraxis der PCIA in anästhesiologischen Abteilungen geben [6]. Der Rücklauf von 25 % der Angeschriebenen war vergleichbar mit anderen Befragungen [9, 12]. Als wichtige Einschränkung und typisch für Umfragedaten können Verzerrungen durch einen Selektionsfaktor sowohl in die eine als auch in die andere Richtung nicht ausgeschlossen werden. Gerade die Angaben zu Komplikationen sind vermutlich durch individuelle und retrospektive Einschätzung geprägt und ermöglichen keine absoluten Aussagen. Die Daten zur Versorgungspraxis in Deutschland sowie zur Häufigkeit potenziell vital bedrohlicher Komplikationen könnten dadurch sowohl über- als auch unterschätzt werden. Dennoch ermöglichen die Ergebnisse eine Orientierung zur aktuellen Praxis und zeigen, dass Sicherheit und Überwachung der PCIA klinisch relevante Themen für das innerklinische Schmerzmanagement sind.

## Behandlungskonzept

Die klinische Einführung der PCIA in den 1970er-Jahren war mit einem Umdenken in der analgetischen Versorgung der Patienten verbunden. Durch eine PCIA kann der Patient (in bestimmten Grenzen) selbst entscheiden, wann und wie häufig ein Opioid appliziert wird. Die PCIA ermöglicht es den Patienten, die Dosis der Schmerzmedikation an den individuellen Bedarf anzupassen, und ermöglicht so und unabhängig vom Pflegepersonal und von ärztlichen Anordnungen einer Bedarfsmedikation, seinen Schmerz selbst kontrollieren. Der Patient ist damit aktiv in die Therapie eingebunden und hat ein gewisses Maß an Autonomie. Dieser Aspekte sind heutzutage hochaktuell und werden wird nach wie vor für moderne medizinische Therapiekonzepte sowie die Beurteilung des Erfolgs von Therapien gefordert [28, 31]. Durch die PCIA-Technik wurde erstmalig auch deutlich, wie unterschiedlich der postoperative Analgetikabedarf sein kann.

Ob die PCIA heutzutage anderen systemischen Applikationsformen überlegen ist, ist hinsichtlich der Studienlage unklar. Eine Cochrane-Analyse zur PCIA vs. alternativer systemischer Opioidgabe umfasst 49 bis 2015 publizierte Studien mit 3412 Patienten [17]. Bei etwas höherem Opioidverbrauch (7 mg in den ersten 24 h) waren die Schmerzintensität unter PCIA niedriger, die Patientenzufriedenheit deutlich höher und Nebenwirkungen etwa gleich häufig. Die meisten Untersuchungen hierzu stammen jedoch aus Zeiten, in denen wenige Alternativen bezüglich des Applikationswegs zur Verfügung standen. Viele dieser randomisierten kontrollierten Studien verglichen die Effektivität der PCIA mit der i.m.-Morphin-Applikation, welche heute obsolet ist [4]. Auch zeigten sich Schwächen im Studiendesign. In mehreren Studien mit positiven Effekten der PCIA stand den Patienten mit diesem Verfahren eine höhere äquipotente Opioiddosis als der Kontrollgruppe zur Verfügung [24]. Mittlerweile ist ein breites Spektrum an oralen und retardierten Opioiddarreichungsformen verfügbar, die sich in den letzten Jahren vermehrt in der perioperativen Akutschmerztherapie etablierte haben [9, 11]. Einige wenige Studien zum Vergleich Opioide als PCIA oder als orale Medikation in der Geburtshilfe zeigten keinen Vorteil eines Verfahrens hinsichtlich Schmerzintensität und Patientenzufriedenheit [7, 22]. Neuere Untersuchungen mit einem sublingualen System schneiden im Vergleich zur PCIA gleichwertig ab und sind auch in Deutschland mittlerweile etabliert [27].

Da es sich bei der vorliegenden Umfrage um eine Anwenderbefragung handelt, wurden Aspekte der Effektivität, Schmerzlinderung und Zufriedenheit aus Sicht der Patienten nicht erfasst. Somit können wir keine Aussagen zum Vergleich zwischen PCIA und Regionalanästhesie treffen, die in einigen Studien eine höhere Zufriedenheit und Schmerzfreiheit erzielten als die PCIA [17].

Unsere Ergebnisse zeigen zudem einen Wandel des Konzepts. In einigen Kliniken wird die PCIA regelmäßig mit retardierten oralen Opioiden kombiniert. Dies lässt vermuten, dass sich die Vorgehensweise bei der PCIA-Therapie der frühen Jahre in der Praxis verändert hat, und dass die PCIA inzwischen konzeptionell oft stärker im Sinne der patientenkontrollierten „Bedarfsmedikation“ oder als zusätzliches „On-Demand-Verfahren“ genutzt wird als dazu, allein einen bedarfsadaptierten Wirkspiegel aufrechtzuerhalten [9, 11]. In der Bewertung eines solchen Konzepts bestehen in der Autorengruppe unterschiedliche Standpunkte, hingegen Einigkeit, dass höhere Überwachungsstandards für ein solches Vorgehen notwendig sind.

Wissenschaftliche Evidenz zu Sicherheit und Wirksamkeit dieses Vorgehens besteht bisher nicht. Aussagekräftige Untersuchungen zum Risiko zentralnervöser Nebenwirkungen bei Kombination der PCIA mit retardierten Opioiden fehlen. Die Ergebnisse der vorliegenden Befragung zeigen, dass in den Kliniken, in denen regelmäßig retardierte Opioide mit einer PCIA-Therapie kombiniert wurden, auch häufiger vitale Komplikationen erfasst wurden. Dies kann in unserer Befragung aber auch darauf zurückzuführen sein, dass durch die intensivere Überwachung und die höhere Verfügbarkeit eines Akutschmerzdienstes Komplikationen häufiger erfasst wurden. Fakt ist aber auch, dass diese Komplikationen trotz Überwachung berichtet wurden. Kritisch ist anzumerken, dass wir nur allgemein nach Kombination PCIA plus orales Opioid gefragt haben. Dadurch bleibt unklar, in welchem Gesamtkonzept die Kombination in den antwortenden Klinken tatsächlich erfolgte, z. B. von vorneherein bei allen Patienten, bei Patienten mit evidentem oder absehbar erhöhtem Opioidbedarf oder zum Ausschleichen/Überlappen der PCIA-Therapie auf ein orales Konzept. Für zukünftige Untersuchungen sollte dies jedoch spezifiziert werden. Auch Studien zur Sicherheit der PCIA bei paralleler Gabe oraler retardierter Opioide sind notwendig. Publikationen zu Zwischenfällen bei kontinuierlicher Applikation lassen vermuten, dass eine Teilgruppe mit geringem Opioidbedarf gefährdet ist bzw. die basale Applikation oft mit unverhältnismäßig hohen kontinuierlichen Dosen erfolgte [21].

Für die kontinuierliche i.v.-Opioid-Applikation auf Normalstationen wurde im Vergleich zu einer bolusgesteuerten PCIA ein 5‑fach erhöhtes Risiko einer Atemdepression beschrieben [21, 30]. Eine Basalrate sollte außerhalb von Überwachungseinheiten bei einem „Standardpatienten“ nicht eingesetzt werden [19]. In 4 % der Kliniken kommt dennoch standardmäßig (in 25 % in Ausnahmefällen) auf Normalstationen eine Basalrate in Kombination mit den patientengesteuerten Boli zum Einsatz. Dies entspricht in etwa den Zahlen, die im Rahmen des Akutschmerzzensus 2012 erhoben worden sind (Basalrate auf Normalstation: 7 % in Krankenhäusern ohne ASD, in Krankenhäusern mit ASD: 5 %) [9, 11].

## Indikationen und Kontraindikationen

Das Indikationsspektrum für eine PCIA hat sich seit ihrer Einführung in die klinische Praxis deutlich verschoben. Galt sie früher nach größeren operativen Eingriffen als Standard, ist ihr Einsatz nach Etablierung der verschiedenen Techniken der Regionalanalgesie scheinbar deutlich in den Hintergrund getreten, oftmals eher ein Verfahren der 2. Wahl. Für zwei Drittel der Kliniken wurden als primäre Indikation für eine PCIA-Therapie Kontraindikationen oder das Versagen eines regionalanästhesiologischen Verfahrens angegeben. Lediglich in einem Drittel der Kliniken wurde die PCIA als primäres Analgesieverfahren bei ausgewählten Operationen eingesetzt. Im nichtoperativen Bereich und bei Kindern wurde die PCIA deutlich seltener eingesetzt. In dieser Erhebung zeigte sich eine eher geringe Zahl an Kliniken, bei denen eine PCIA auch bei Kindern unter 15 Jahren angewandt wurde [20]. Es wurde jedoch nicht erfasst, ob und in welchem Umfang Kinder durch die befragten anästhesiologischen Abteilungen versorgt wurden.

Einige der befragten Kliniken schlossen Patienten mit einem erhöhten Risiko für eine Atemdepression aus. Hier standen Adipositas, obstruktives Schlafapnoesyndrom (OSAS) und chronische obstruktive Lungenerkrankungen im Vordergrund. Leitlinien und evidenzbasierte Empfehlungen hierzu sind heterogen. In der *S3-Leitlinie „Behandlung akuter perioperativer und posttraumatischer Schmerzen“* von 2007 sind keine Kontraindikationen für die Anwendung der PCIA enthalten [19]. Die australische und neuseeländische Evidenzsammlung *Acute Pain Management: Scientific Evidence* betont das kumulierte Risiko bei Patienten mit OSAS, Adipositas und Opioidmedikation [29]. Nach Einschätzung von Faßbender et al. sollten OSAS-Patienten unter Opioidtherapie (je nach Konstellation mit oder ohne CPAP-Behandlung) stets mit Monitorkontrolle per Telemetrie oder intensivmedizinisch überwacht werden [13]. Sowohl zu Kontraindikationen als auch zum risikoadaptierten Monitoring einer PCIA fehlen aktuelle Empfehlungen für Deutschland. Die Vereinbarungen zwischen chirurgischem und anästhesiologischem Berufsverband zur Schmerztherapie chirurgischer Patienten enthalten keine Empfehlung zur PCIA, hingegen eine klare Aussage für Regionalanästhesieverfahren, die in Krankenhäusern ohne ASD nur in Überwachungsbereichen betreut werden sollen [15].

## Patientensicherheit und Überwachungsstandards

Von den angegebenen Vorkommnissen im Kontext der PCIA wären möglicherweise einige durch entsprechende Klinikstandards und Schulungen vermeidbar gewesen. Von den 335 berichteten, potenziell vital bedrohlichen Komplikationen hatte der Großteil keine langfristigen Folgen. Immerhin 58 Fälle erforderten eine intensivmedizinische Behandlung, und 3 Komplikationen gingen mit bleibenden Schäden einher. Ein Fall verlief nach den Angaben im Fragebogen tödlich. Eine Analyse, inwieweit die PCIA bzw. das Opioid im direkten kausalen Zusammenhang mit diesen Komplikationen stand, und ob evtl. weitere Faktoren dazu beitrugen, wäre sicherlich aufschlussreich, war im Rahmen dieser Umfrage jedoch nicht möglich. Auch wenn eine solche Einschätzung nur mit erheblicher Unschärfe erfolgen kann, liegt die geschätzte Rate aller angegebenen vitalen Komplikationen (Bezug zur angegebenen Gesamtzahl durchgeführter PCIA-Therapien; konkrete und geschätzte Angaben zusammen) mit 0,69 % unter den Angaben aus der Literatur [4, 26].

Das Auftreten von „Atemdepressionen“ unter PCIA-Therapie lag in einer Analyse aller bis 1999 publizierten Studien je nach Überwachungsmodus zwischen 1 % und 12 % (1 % bezogen auf die Reduktion der Atemfrequenz je nach Studie unter 8 bzw. 10/min, 12 % bezogen auf Reduktion der Sauerstoffsättigung unter einem für die jeweilige Studie gewählten „Cut-off“-Wert in der Pulsoxymetrie) [4]. Zum Vergleich mit anderen Verfahren ergaben sich für i.m.-Injektionen von Opioiden eine Rate bis zu 37 % und bei Epiduralanalgesie bis zu 15 % [4]. In diesen Studien wurden Patienten jedoch gezielt überwacht. Trotz Studienbedingungen spiegelt sich auch hier der Zusammenhang zwischen der Art des Monitorings und der Komplikationssensitivität wider.

Die Standards hinsichtlich der Überwachungsmodi von Vitalparametern unterschieden sich zwischen den Kliniken erheblich, und höhere Überwachungsstandards gingen mit vermehrter Wahrnehmung von vital bedrohlichen Komplikationen einher – eine Assoziation, die schon zuvor beschrieben wurde [32]. Es ist unwahrscheinlich, dass bei niedrigem Überwachungsstandard weniger Komplikationen auftreten. Hier kann aufgrund des retrospektiven Charakters der Studie nur vermutet werden, dass in Krankenhäusern mit niedrigerem Überwachungsstandard die Dunkelziffer hoch ist. Allerdings sind auch andere Zusammenhänge, wie z. B. eine erhöhte Überwachungspraxis in Kliniken mit kränkeren Patienten, die eine PCA erhalten, möglich.

Es ist keinesfalls Intention dieser Arbeit, die PCIA als Verfahren infrage zu stellen. Vielmehr soll verdeutlicht werden, dass es sich – wie bei anderen Verfahren der Akutschmerztherapie auch – um ein Verfahren mit potenziellen Risiken handelt, welches in die Hände von erfahrenen Behandlern gehört und struktureller Voraussetzungen bedarf. Die US-amerikanischen Fachgesellschaften für Anästhesiologie und Schmerzmedizin empfehlen, Patienten mit systemischer Opioidapplikation grundsätzlich anhand von Sedierungsgrad, respiratorischem Status und von Nebenwirkungen zu überwachen [5, 16]. Auch in Deutschland sollte dies im Sinne der Patientensicherheit einen hohen Stellenwert haben [2, 19]. Eine aus Sicht der Autoren wesentliche Voraussetzung hierzu ist ein Akutschmerzdienst, der die Strukturempfehlungen der Deutschen Gesellschaft für Anästhesiologie und Intensivmedizin e. V. (Nürnberg) erfüllt. Von den Krankenhäusern, die die PCIA einsetzten, verfügten knapp 80 % über einen ASD, ein mit früheren Befragungen vergleichbares Ergebnis [9, 11, 18]. Dabei wurde jedoch in der vorliegenden Untersuchung die Heterogenität der ASD hinsichtlich ihrer Qualität nicht berücksichtigt [10]. Dort, wo vorhanden, oblag dem ASD meistens die Verantwortlichkeit für die PCIA, teilweise auch für die zusätzliche Erfassung von Vitalparametern. Bedenklich erscheint, dass in 14 % der antwortenden Kliniken die PCIA eingesetzt wurde, ohne dass es einen ASD zur Betreuung gab.

## Fazit für die Praxis

Die patientenkontrollierte intravenöse Analgesie (PCIA) ist ein häufiges und in vielen Krankenhäusern eingesetztes Analgesieverfahren mit einer heterogenen Anwendungs- und Überwachungspraxis. Unerwünschte Vorkommnisse und potenziell vital bedrohliche Komplikationen wurden von einem relevanten Anteil der Kliniken berichtet. Deren Detektion scheint mit Überwachung von zusätzlichen Vitalparametern, über die der normalen Stationsstandards hinaus, verbessert zu sein. Viele der berichteten Probleme unterliegen der Kontrolle durch das medizinische Personal und sind damit potenziell vermeidbar. Eine sorgfältige Patientenauswahl, Standards zur Anwendung der PCIA und deren Monitoring, ein Akutschmerzdienst sowie gut geschultes Stationspersonal stellen grundlegende Voraussetzungen für eine verantwortungsvolle PCIA-Therapie dar. Aktuelle Empfehlungen zu sicherer Anwendung und Überwachung der PCIA-Therapie fehlen jedoch. Die Ergebnisse sollten Anlass geben, im Sinne des Risikomanagements aktuelle Behandlungs- und Überwachungsstandards zu erarbeiten.
